# Supplementary material for: Dynalign II: common secondary structure prediction for RNA homologs with domain insertions
Source: Nucleic Acids Res. 2014 Nov 21;42(22):13939–48. doi: 10.1093/nar/gku1172 (PMC4267632; doi:10.1093/nar/gku1172)
Supplement: SUPPLEMENTARY DATA [file supp_gku1172_nar-02021-z-2014-File012.zip › manual/GUI/html/Whats_New.html]

RNAstructure GUI Help -- What's New


|  |  |  |
| --- | --- | --- |
|  | RNAstructure GUI Help What's New | - Contents - Index |
| Version 5.6 (Released 7/31/2013)   1. The Windows GUI now runs in parallel on multicore computers for predicting lowest free energy structures, calculating partition functions, calculating free energy changes, oligoscreen calculations, and stochastic sampling. 2. Fixed a bug with reading HMM alignment parameters for Dynalign calculations in the Windows GUI. This was causing the program to crash for Dynalign calculations.   Version 5.5 (Released 4/5/2013)   1. Fixed bug in JAVA GUI where opening .seq files from disk would sometimes fail.   Version 5.4 (Released 8/2/2012)   1. Added TurboKnot function to TurboFold module. 2. JAVA GUI overhauled to appear more native-like on Mac and Linux. 3. Windows GUI updated to Windows 7 standards. 4. Breaking DNA pseudoknots added to JAVA GUI. 5. Removed "New Window" and "Arrange Icons" options, which did nothing, from the menus of the Windows GUI. 6. Corrected bug in JAVA GUI where the partition function bimolecular module sometimes overwrote its second input sequence file, rather than using the desired output name. 7. Corrected bug in JAVA GUI where file chooser extension sanity checks sometimes failed. 8. Fixed a bug in the Windows GUI, where pressing the F1 key resulted in a "Failed to Launch Help" window in Windows XP and later.   Version 5.3 (Released 5/10/2011)   1. Added TurboFold module for RNA folding. 2. Fixed bug in JAVA version that occassionally prevented drawing of suboptimal structures. 3. Fixed a bug where bimolecular partition functions were missing some states (single dangling ends and no dangling ends) at helix ends. 4. Fixed bug in JAVA version that caused OligoWalk energy values to sometimes be displayed incorrectly. 5. Fixed bug in partition function calculations when SHAPE data are used where the precision of the SHAPE pseudo free energy changes was truncated. 6. Corrected OligoWalk to show the correct temperature of folding free energy changes when user adjusts the temperature of the calculation. (Prior to this, free energy changes were always listed as being at 37 degrees C.) 7. Corrected bug in JAVA version where the message bar at the bottom of the screen didn't always update when a temperature menu was clicked. 8. Corrected bug in JAVA version in OligoScreen where file names were being refreshed incorrectly. Also switched oligomer type default to DNA, rather than RNA.- Corrected bug in      JAVA version of OligoWalk where oligonucleotides were always RNA, even when DNA was selected.    Version 5.2 (Released 11/17/2010)   1. Added Multilign to interface. 2. Added color annotation for single-stranded nucleotides. See the draw help page. 3. Added support for writing ct files for long sequences (>9,999 nucleotides). 4. Removed GAIL rule from dnamiscloop.dat and dnamiscloop.dh. 5. Made the internal loop asymmetry penalty a single constant for DNA folding. 6. On Windows only, changed the minimum helix length in ProbKnot from 2 to 3, which is the preferred value. 7. On Windows only, fixed the Dynalign "Alignment File" button to display .ALI files by default. 8. In Dynalign, revised the model for using alignment data to limit the extent of calculation. This can lead to a different set of suboptimal structures than previous versions. This also fixes a traceback error encountered in rare circumstances. 9. Improved the fonts in this online manual so that text appears better on Windows.    Version 5.1 (Released 6/30/2010)   1. The RNA free energy parameter for adding additional helices to a multibranch loop was changed from -0.9 to -0.6 kcal/mol. This value is that found by linear regression to experimental data. The -0.6 kcal/mol value results in higher average accuracy when using the maximum expected accuracy methods, MEA and ProbKnot. Note that this means that predicted structures may not be identical to those predicted by earlier versions of the software. 2. Menu item names harmonized between Windows GUI and JAVA GUI. 3. Circular drawings for structures with pseudoknots added to Draw module. 4. Improved online help. 5. Fixed a glutch in JAVA version where SHAPE files could not be read from disk. 6. Added the ability to read FASTA-formatted sequence files.    Version 5.03 (Released 3/22/2010)   1. Fixed glitch where failure to enter a title in the New Sequence window resulted in invalid sequence files.    Version 5.02 (Released 1/22/2010)   1. No changes to the GUI.    Version 5.01 (Released 12/30/2009)   1. Changed the default parameters for SHAPE data pseudo free energy changes to match best practices.    Version 5.0 (Released 9/30/2009)   1. Program is now portable across multiple operating systems. 2. Created "RNA" and "DNA" menus to aim specific calculations at particular nucleic acid types. 3. The option box which allowed the user to write thermodynamic details files for Energy Function 2 (Efn2) was non-functional in version 4.6. This has been corrected. 4. Standardized viewing options for drawing and zooming structures and dot plots.    Version 4.6 (Released 5/5/2008)   1. Added the ability to specify SHAPE data for RNA Fold Single Strand. 2. Added stochastic sampling.    Version 4.5 (Released 4/9/2007)   1. Added the capability to specify the maximum distance between paired nucleotides. 2. Added HMM alignment method to limit Dynalign's search space in the alignment domain.    Version 4.4 (released 8/17/2006)   1. Generate All Suboptimal Structures module added. 2. Secondary structures can now be predicted at temperatures other than 37 degrees Celsius. 3. Some menu options in force were grayed out and non-functional in 4.3. This has been corrected. 4. The free energy table for multibranch loops used by Dynalign (dynalignmiscloop.dat) incorrectly used an older set of parameters. This has been corrected. (Note that this was not the case for the Unix source code version of Dynalign, so the two versions are now synchronized.)    Version 4.3 (released 3/16/2006)   1. Base pair probabilities, as determined by the partition function calculation, can now be output to a text file. 2. Dynalign is greatly accelerated by not considering base pairs that are determined by single sequence secondary structure prediction to only occur in structures with relatively high folding free energy changes. Dynalign is also accelerated by redefining the M parameter that limits the alignments that are considered. A much smaller M can now be used. [Note that as of RNAstructure 4.5, the use of M is deprecated.] | | |
| Visit The Mathews Lab RNAstructure Page for updates and latest information. | | |
